# Enhancing the Visibility of Multiple Sclerosis Lesions using Fused Images of Fluid Attenuated Inversion Recovery (FLAIR) and white matter Attenuated Inversion Recovery (WAIR) MRI Sequences

**DOI:** 10.2174/0115734056437290260309135652

**Published:** 2026-04-08

**Authors:** Khalid Alanazi, Abdullah Abujamea, Muhammad Fahim Amjad, Almutairi Fahad, Ali Abdu

**Affiliations:** 1 Department of Radiology and Medical Imaging, King Saud University Medical City, Riyadh, Saudi Arabia; 2 Department of Radiology and Medical Imaging, College of Medicine, King Saud University, Riyadh, Saudi Arabia; 3 Department of Radiology, King Khalid Hospital, Tabuk, Saudi Arabia

**Keywords:** Multiple sclerosis, MRI, FLAIR, White matter attenuated inversion recovery, lesion detection, DIR, MinPE

## Abstract

**Introduction:**

Multiple sclerosis (MS) is a chronic autoimmune disease of the central nervous system in which accurate lesion detection is essential for diagnosis and follow-up. Although the Fluid Attenuated Inversion Recovery (FLAIR) MRI sequence is routinely used, it may underestimate lesions in certain brain regions. This study evaluated whether fused images generated using minimum pixel value extraction (MinPE) from FLAIR and white matter–attenuated inversion recovery (WAIR) sequences improve lesion detection.

**Materials and Methods:**

This retrospective single-center study analyzed brain MRI examinations from 65 patients with suspected or confirmed MS. Imaging protocols included conventional FLAIR and MinPE FLAIR/WAIR images. Two experienced neuroradiologists, blinded to clinical data, independently identified and classified lesions. Lesion detection rates were compared using chi-square analysis, and interobserver agreement was assessed with Cohen’s kappa.

**Results:**

MinPE FLAIR/WAIR images showed improved lesion conspicuity and significantly higher detection rates compared with conventional FLAIR, particularly in the brainstem. Detection was markedly increased in the midbrain, pons, and medulla (*p* ≤ 0.0004), with additional improvement observed in the cerebellar hemispheres (*p* < 0.05).

**Discussion:**

While advanced sequences such as Double Inversion Recovery (DIR) and Phase-Sensitive Inversion-Recovery (PSIR) enhance lesion detection, their longer acquisition times limit routine use. The MinPE FLAIR/WAIR technique improves lesion visibility in challenging regions with minimal impact on scan time, allowing a more accurate estimation of disease burden.

**Conclusion:**

MinPE FLAIR/WAIR post-processing enhances MS lesion detection and may represent a practical addition to routine MRI protocols.

## 1 INTRODUCTION

Multiple sclerosis (MS) is a chronic autoimmune inflammatory disorder of the central nervous system (CNS) characterized by demyelination, axonal damage, and neurodegeneration [1-3]. Approximately 2.8 million individuals are affected by MS worldwide [4]. It occurs predominantly in the 20-40 age range, and it affects women more often than men [2, 5, 6]. A hallmark feature of MS pathology is the formation of focal inflammatory lesions in the CNS located in the brain and spinal cord, mostly within the White Matter of the CNS [7, 8]. The inflammatory process causes interference in the normal transmission of signals along the nerve axons, which may give rise to a variety of neurological symptoms, including fatigue, altered sensation, reduced motor function, and cognitive impairment [9-11]. MRI plays an important role in diagnosing and managing patients with MS [10, 12]. Typical MRI sequences used to identify and characterise the location, extent, and morphology of MS lesions are T1, T2-weighted sequences, and Fluid Attenuated Inversion Recovery (FLAIR) sequences. [13, 14] FLAIR MRI sequences show great sensitivity for demonstrating the presence of periventricular and juxtacortical lesions, which are commonly seen in patients with MS. [10, 13, 15].

Although conventional MRI has played an essential role in detecting multiple sclerosis (MS) lesions, challenges exist with identifying and differentiating MS lesions using MRI [16]. The traditional fluid attenuated inversion recovery (FLAIR) sequence may have limited sensitivity for certain lesions, including cortical and subcortical lesions, which are increasingly recognized as important contributors to MS pathology. [16 -18] Additionally, distinguishing MS lesions from other white matter abnormalities, such as vascular lesions and nonspecific hyperintensities, can lead to delays in identifying the correct clinical diagnosis and affect how patients are managed [16, 19].

**Fig. (1):**
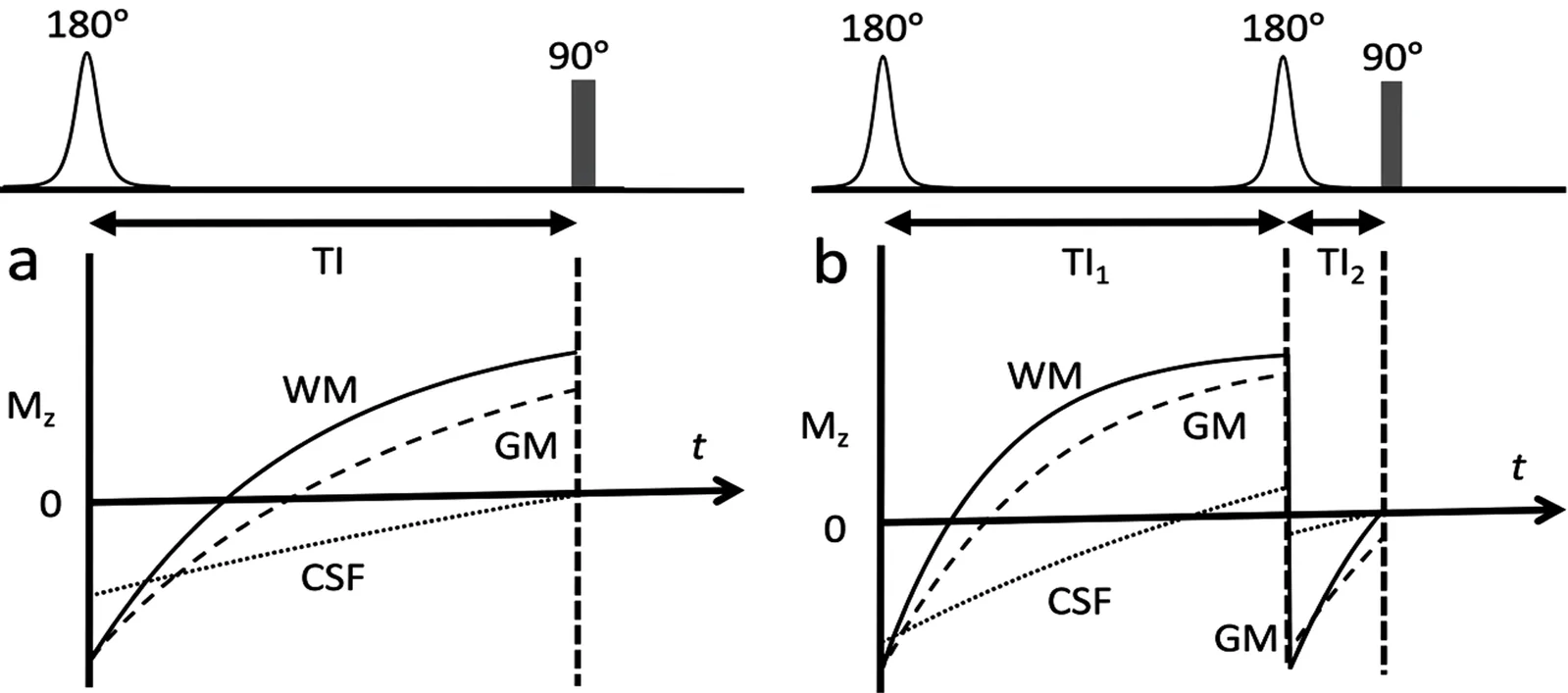
Pulse sequence diagram of inversion recovery (IR) (**a**) and double inversion recovery (DIR) (**b**).

To overcome these challenges, advanced MRI techniques have been developed to enhance the detection and characterization of MS lesions. One of these advanced MRI techniques is the double inversion recovery (DIR) sequence, which will permit the selective suppression of signals from both cerebrospinal fluid (CSF) and the brain's white matter (WM), which will result in a better contrast between the lesion and the background image of the brain [16, 17, 20, 21]. However, DIR is limited by its relatively long acquisition time (an average of 7 minutes depending on the technique and parameters) and its lower signal-to-noise ratio (SNR) compared with the conventional FLAIR sequence, as the multiple 180° inversion pulses reduce the overall SNR of the image (Fig. **1**) [22-25].

To address these limitations, we explored the use of fused images combining FLAIR and WAIR sequences, referred to as “MinPE FLAIR/WAIR” as a potential tool for improving the detection of MS lesions. By integrating complementary information from the FLAIR sequence, which suppresses the signal from CSF, and the WAIR sequence, which suppresses the signal from white matter, the “MinPE FLAIR/WAIR” method offers the potential for improved lesion conspicuousness and diagnostic accuracy in MS [22, 26, 27]. However, its clinical utility in routine practice remains to be fully established, and further research is needed to validate its efficacy and clarify its role in the diagnostic workup of MS [16, 28].

This study aimed to assess the diagnostic value of fused images generated using Minimum Pixel Value Extraction of FLAIR and WAIR sequences “MinPE FLAIR/WAIR”, in improving the detection of multiple sclerosis (MS) lesions compared with the conventional FLAIR sequence. Our findings demonstrate that MinPE FLAIR/WAIR provides greater detection rates for MS lesions in the cortical, juxtacortical, periventricular, and subcortical regions than the conventional FLAIR sequence.

## 2 MATERIALS AND METHODS

### 2.1 Study Design

A retrospective observational study was conducted to evaluate the effectiveness of fused Fluid-Attenuated Inversion Recovery (FLAIR) and white-matter-attenuated inversion recovery (WAIR) sequences (referred to as “MinPE FLAIR/WAIR”) in improving the detection of multiple sclerosis (MS) lesions. The study was performed at a single center, and the data were obtained from the institution’s MRI database. Ethical approval for this study was obtained from the Institutional Review Board of the College of Medicine, King Saud University (Ref. No. 23/0315/IRB), and the requirement for patient consent was waived because of the retrospective study design. This article was reported in accordance with the STARD Reporting Checklist.

### 2.2 Study Population

This study enrolled 65 patients with multiple sclerosis (MS) who underwent brain MRI for suspected or confirmed MS at King Khalid University Hospital, Riyadh, Saudi Arabia, between January 2021 and December 2023. Using the Connors method for post-hoc sample size estimation and comparing our sample size to other similar studies, both methods established that about 63 subjects would be the minimum number of patients needed to test sufficient statistical power for detecting any difference in lesion detection rate. As a result, our final sample of 65 patients satisfied this requirement. Eligible patients were aged ≥ 18 years and had a confirmed diagnosis of MS based on the 2017 McDonald criteria [29]. The following conditions precluded patients from participating: incomplete imaging data; poor imaging quality; comorbid conditions that might negatively affect MRI analysis, such as severe atrophy of the brain; significant cerebrovascular disease; and neurological disorders.

**Fig. (2):**
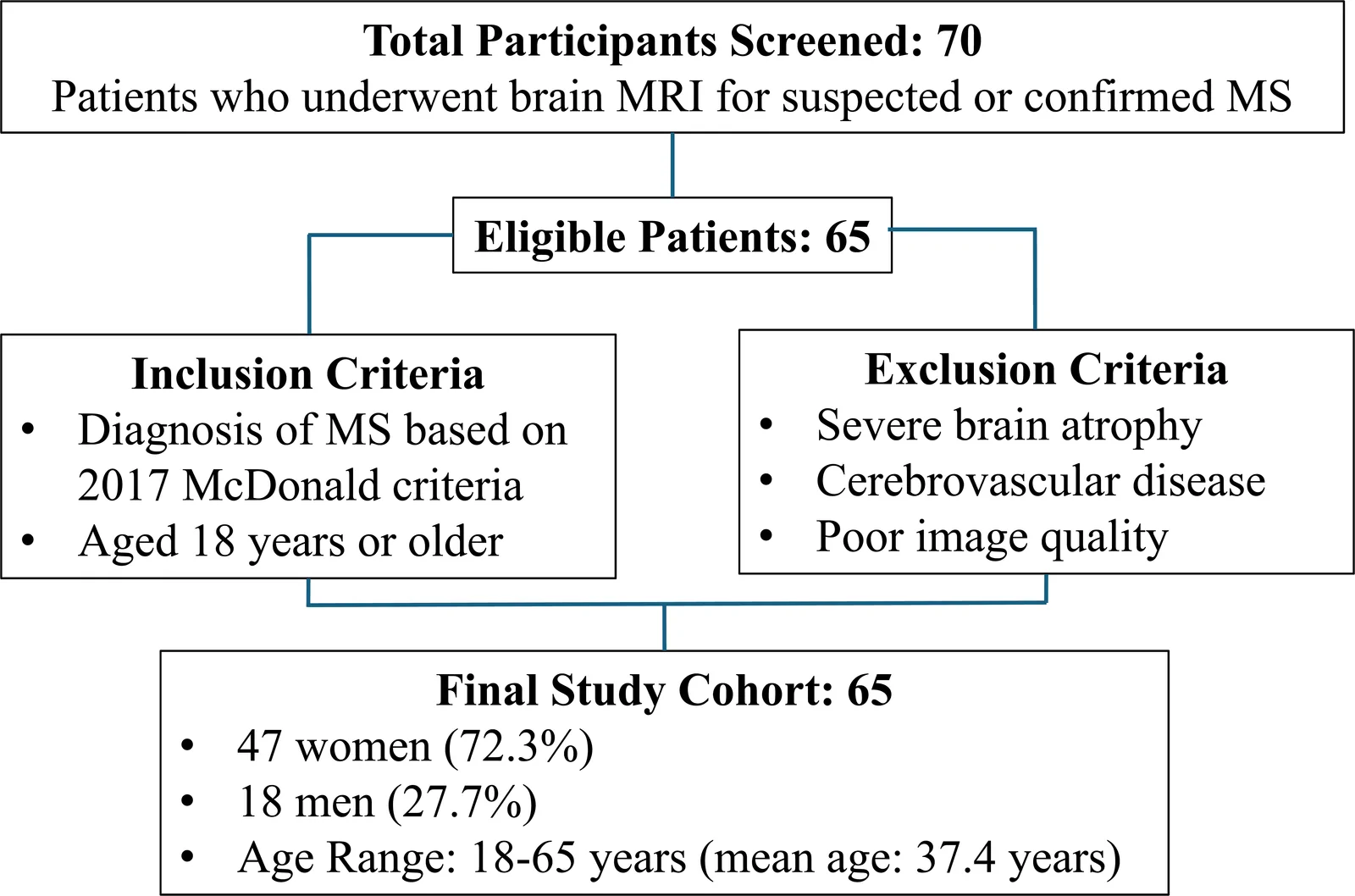
Selection process of the study.

After applying these criteria, 65 patients met the inclusion requirements; five were excluded because of severe motion artifacts. The final cohort was predominantly female, comprising 47 women (72.3%) and 18 men (27.7%) (Fig. **2**).

### 2.3 Cohort Characteristics

The mean age of the patients was 37.4 years (range, 18–65). The mean duration of MS since diagnosis was 7.5 years (range, 1–30). The cohort included the following MS subtypes: relapsing–remitting MS (RRMS), 40 patients (61.5%); secondary progressive MS (SPMS), 18 patients (27.7%); and primary progressive MS (PPMS), seven patients (10.8%). The mean Expanded Disability Status Scale (EDSS) score was 3.2 (range, 1.0–8.5), indicating mild-to-moderate disability. Patients received various disease-modifying therapies (DMTs), including interferon-beta (n = 25, 38.5%), glatiramer acetate (n = 15, 23.1%), natalizumab (n = 10, 15.4%), or no treatment (n = 15, 23.1%).

### 2.4 Imaging Protocol

All MRI scans were acquired using a standard 12-channel head coil. The imaging protocol included conventional axial FLAIR and WAIR sequences. The total scan time was 2:29 min for the FLAIR sequence and 1:52 min for the WAIR sequence, giving a combined acquisition time of approximately 4 min and 21 s. This is notably shorter than the approximately 7 min required for a DIR sequence, which is known for its superior lesion detection through selective suppression of white matter and cerebrospinal fluid signals. The parameters used for the FLAIR sequence were as follows: repetition time (TR) of 8000 ms, echo time (TE) of 95 ms, inversion time (TI) of 2750 ms, field of view (FOV) of 230×230 mm, slice thickness/spacing of 5/1 mm, an image matrix of 320/224, echo train length (ETL) of 12, and one signal average. The total scan time was 2:29 minutes, while the parameters used for the WAIR sequence were as follows: repetition time (TR) of 6000 ms, echo time (TE) of 52 ms, inversion time (TI) of 300 ms, field of view (FOV) of 230×230 mm, slice thickness/spacing of 5/1 mm, an image matrix of 320/224, echo train length (ETL) of 12, and one signal average, with a total scan time of 1:52 minutes. When fusing two image sequences, the source images must share the same spatial resolution to ensure accurate voxel-wise integration. In practice, any spatial resolution may be used, provided that it is consistent across both sequences before fusion.

### 2.5 Image Analysis

The MinPE filter is an image processing technique based on minimum pixel-wise operations used to combine images. This operation takes two or more input images and generates an output image in which the value of each pixel is the minimum of the corresponding pixels from all input images. Mathematically, for two input images, A and B, the minimum pixel-wise operation is represented as:

C(x, y) = min(A(x, y), B(x, y))

where (C(x, y)) is the output pixel at coordinates (x, y), and (A(x, y)) and (B(x, y)) are the corresponding pixels in input images A and B, respectively. The minimum operation can be helpful in several image processing applications, such as determining common or overlapping areas between two images, reducing noise by averaging the values from two or more noisy images that are acquired, and creating masks using the results of segmentation on both images. The MinPE FLAIR/WAIR images were generated by applying the minimum function between pixel values of the FLAIR and WAIR sequences of corresponding pixels. Therefore, the resulting pixel values of each MinPE pixel correspond to the minimum value of the two respective pixels (*i.e.*, pixels from FLAIR and WAIR) from the two respective sequences. This operation was performed using a dedicated image combination tool on a GE Advantage Windows workstationV7.1.7. This method is independent of the scanner type because it is fundamentally a post-processing procedure. The workflow relies on standard FLAIR and WAIR images and applies the same coregistration and analysis steps, regardless of the scanner vendor or model. Because all operations occur after image acquisition, the method does not depend on proprietary hardware or pulse-sequence implementations. Therefore, it can be replicated on a scanner as long as the corresponding image contrasts are available.

### 2.6 Coregistration Step

**Fig. (3):**
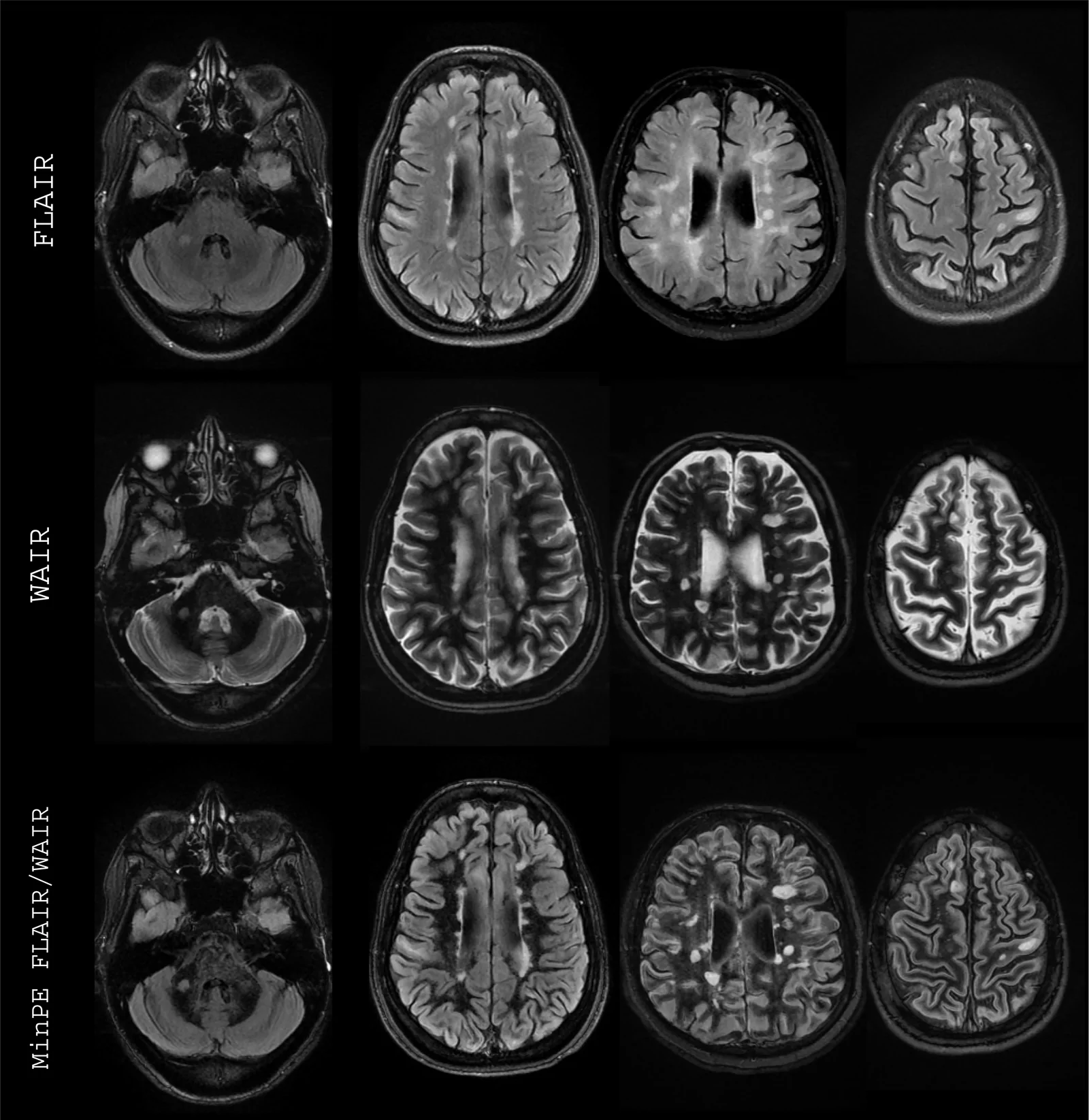
Example of combining FLAIR and WAIR images to generate a MinPE FLAIR/WAIR image. The MinPE FLAIR/WAIR images were generated by fusing the minimum pixel value extraction, which is an operation that consists of finding the minimum image intensity values pixel by pixel in both the FLAIR and WAIR sequences.

Before implementing the MinPE filter, the FLAIR and WAIR images were co-registered to ensure precise alignment. This step was performed using rigid body transformation algorithms in Functool software (version 9.4.05a) on a GE Advantage Windows workstation (v7.1.7) to correct for potential patient motion between the two sequences. Coregistration mitigated misalignment errors, ensuring accurate voxel-to-voxel correspondence between the FLAIR and WAIR images (Fig. **3**). The registration pipeline is fully automated and does not require user-defined preprocessing steps or manual parameter tuning. This automated approach was deliberately selected to reflect routine clinical practice because it allows image fusion to be performed by technologists or radiologists without the need for advanced expertise in image processing. The coregistration quality was assessed using systematic visual inspection, which is a widely used and recommended approach in clinical MRI workflows when rigid-body transformations are applied. For each dataset, we visually evaluated the alignment of the ventricular boundaries, cortical and subcortical white matter interfaces, and consistency of the lesion location across FLAIR and WAIR images. Datasets with visible misalignments were excluded, as shown in Fig. **S1**.

### 2.7 Reader Evaluation and Quality Assessment

Two experienced neuroradiologists independently reviewed all MRI scans and were blinded to the clinical information. The two neuroradiologists had over 10 years of experience working with MRI. They evaluated whether or not MS lesions were present in pre-defined regions of the brain, such as cortical gray matter, juxtacortical white matter, subcortical white matter, periventricular white matter, corpus callosum, basal ganglia, thalamus & internal capsule. To minimize potential reader bias and recall effects, all image datasets were anonymized and assigned numeric identifiers before evaluation. Conventional and fused images were independently presented to the neuroradiologists. Lesion detection was based on a visual assessment. A qualified medical physicist assessed the image quality. Data sets demonstrating poor imaging detail, either caused by severe image artifacts or from motion between FLAIR and WAIR scans, are excluded from this study. To ensure the reliability and validity of the study findings, quality control measures were implemented throughout the data collection and analysis. This included a careful review of MRI scans by trained neuroradiologists for image artifacts and consistent interpretation.

### 2.8 Statistical Analysis

Statistical analyses were performed using the IBM SPSS software (version 26). A priori sample size calculation was not performed because of the retrospective design of the study, which included all eligible MRI scans during the study period. However, a post-hoc power analysis was conducted to estimate the statistical power based on the observed effect size and the available sample of at least 60 patients, yielding a power of 90%. Descriptive statistics were used to summarize patient demographics and lesion characteristics as frequencies, percentages, means, and standard deviations. The agreement between conventional FLAIR and “MinPE FLAIR/WAIR” sequences in lesion detection was assessed using the chi-square test, with Fisher’s exact test applied when more than 20% of cells had an expected count of zero. Statistical significance was set at *p* ≤ 0.05. Inter-observer agreement for lesion detection was evaluated using Cohen’s kappa (κ), which quantifies the degree of agreement between the two readers. Kappa values were interpreted as follows: < 0.20 = poor, 0.21–0.40 = fair, 0.41–0.60 = moderate, 0.61–0.80 = good, and 0.81–1.00 = strong agreement. Post-hoc power analysis was performed based on the observed effect size for the primary comparison between MinPE FLAIR/WAIR and conventional FLAIR lesion detection rates in brainstem regions, where statistically significant differences were identified. Using the observed effect size and sample size, the estimated study power was approximately 90% for detecting differences of this magnitude. This power estimate reflects the ability to detect large effect sizes in anatomically challenging regions and may not be generalizable to supratentorial regions where smaller effect sizes were observed.

## 3 RESULTS

**Fig. (4):**
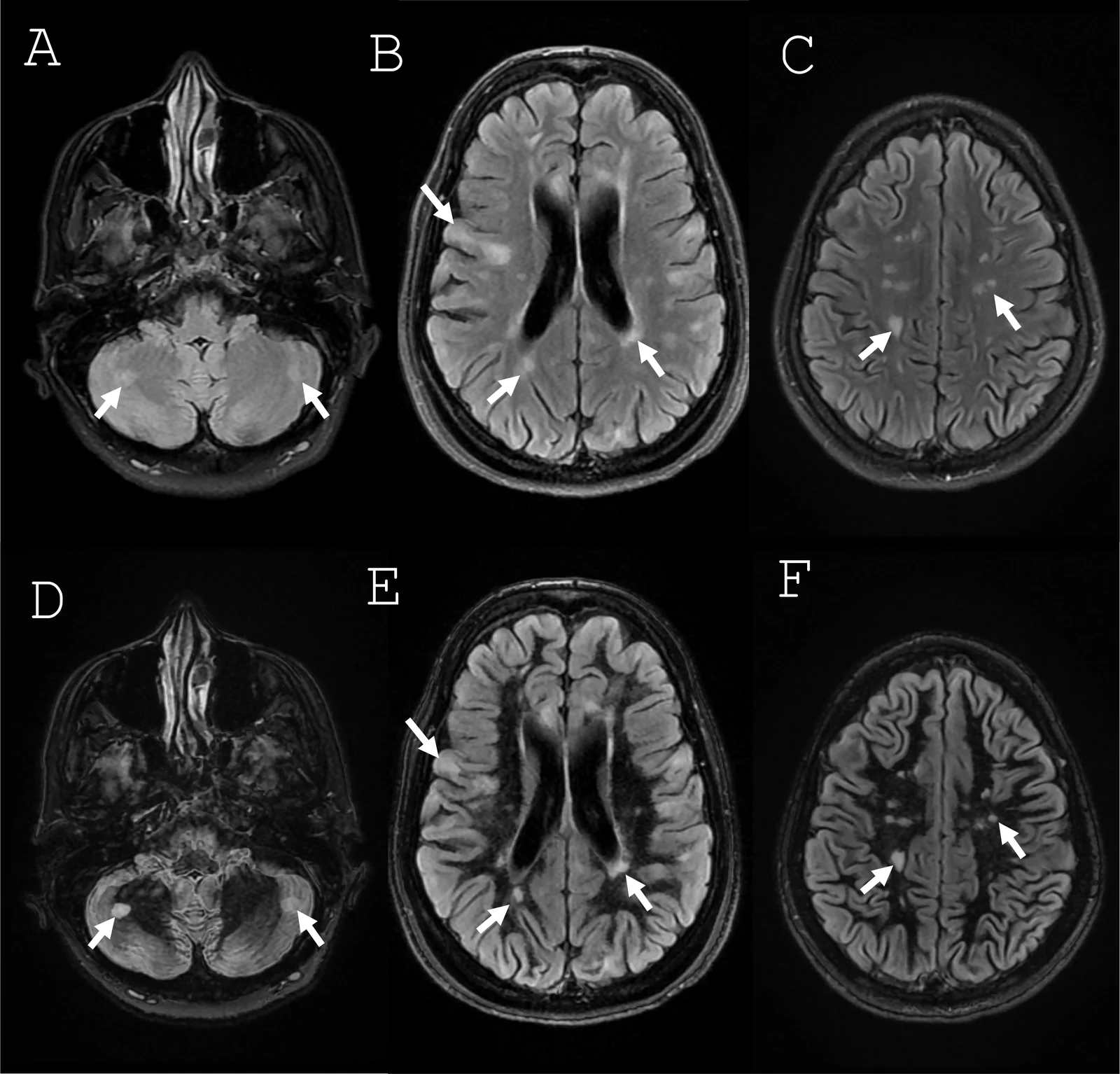
Axial brain MRI at the level of the posterior fossa (**A**&**D**), lateral ventricles (**B**&**E**), and centrum semiovale (**C**&**F**) for three different MS patients. Comparison of FLAIR images in the top row and MinPE FLAIR/WAIR images in the bottom row. Lesions marked with white arrows were brighter and more easily identifiable on MinPE FLAIR/WAIR than on FLAIR.

In general, MinPE FLAIR/WAIR images showed improved detection rates compared with conventional FLAIR across several brain regions (Fig. **4**). Although in supratentorial regions, MinPE FLAIR/WAIR demonstrated higher mean lesion counts compared with conventional FLAIR; however, most of these differences did not reach statistical significance.

Table **1** presents the demographic profiles of the 65 patients, including sex and age distribution. Of the total patients, 18 (27.7%) were male and 47 (72.3%) were female, indicating a higher representation of females in the group. The age distribution showed that 27.7% of patients were under 25 years of age, the largest proportion (35.4%) was between 25 and 30 years of age, 24.6% were between 30 and 40 years of age, and 12.3% were older than 40 years of age. This reflects a relatively young patient cohort, with most patients aged 25–30 years. The overall age range was 17–57 years, with a mean age of 29.96 years and a standard deviation of 2.11, indicating minimal variation around the average.

A comparison between the FLAIR and MinPE FLAIR/WAIR images was performed using the chi-square test. The inter-observer agreement for each imaging method (FLAIR and MinPE FLAIR/WAIR) was assessed using the kappa (κ) statistic. In the cortical gray matter, both observers identified positive findings in 24.6% of FLAIR images, whereas MinPE FLAIR/WAIR showed a slightly higher rate (33.8%). The chi-square test yielded a *p*-value of 0.247, indicating no statistically significant difference between the two methods of anesthesia. The inter-observer agreement was perfect, with κ = 1.00 for both FLAIR and MinPE FLAIR/WAIR. For the juxtacortical white matter, positive findings were observed in 43.1% of the FLAIR images and 46.2% of the MinPE FLAIR/WAIR images. The second observer reported similar rates (43.1% and 46.2%). The chi-square test yielded a *p*-value of 0.724, again showing no significant difference. Kappa values of 0.971 and 0.973 indicated a strong inter-observer agreement. In the subcortical white matter, positive findings were noted in 52.3% of the FLAIR images and 58.5% of the MinPE FLAIR/WAIR images. However, this difference was not statistically significant (*p* = 0.480). The inter-observer agreement remained high, with κ = 0.972 for the first observer and κ = 1.00 for the second, reflecting excellent reliability.

In the periventricular white matter, positive findings were observed in 87.7% of the FLAIR images, increasing slightly to 90.8% with MinPE FLAIR/WAIR. The chi-square test yielded a non-significant *p*-value of 0.571, indicating no meaningful difference between the two methods. The kappa (κ) values were 0.936 and 0.968, reflecting excellent inter-observer agreement between the two observers. In the corpus callosum, the first observer reported positive findings in 12.3% of the FLAIR images, and the second observer reported positive findings in 13.8% of the FLAIR images. MinPE FLAIR/WAIR showed a slightly higher rate (15.4%). The differences between the techniques were not statistically significant (*p* = 0.612 and *p* = 0.804, respectively). The κ value of 0.932 further highlights the high level of agreement between the observers (Table **2**).

**Fig. (5):**
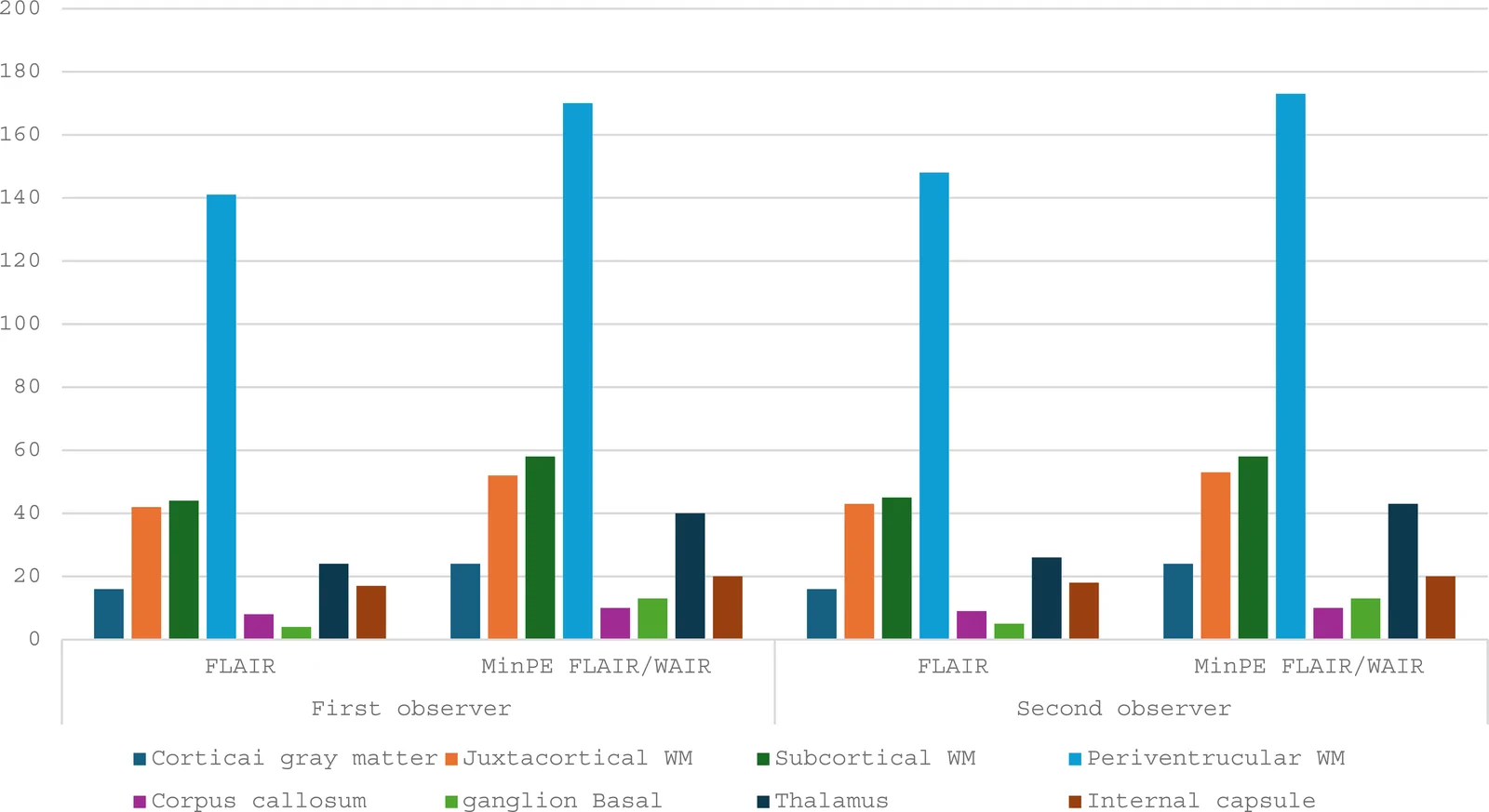
Comparison between the total number of findings in the hemispheric FLAIR and MinPE FLAIR/WAIR regarding the two observers and agreement between the two observers; the y-axis represents the number of detected MS lesions.

In the basal ganglia, positive findings were detected in 6.2% of FLAIR images by the first observer, increasing to 13.8% with the MinPE FLAIR/WAIR protocol. Chi-square testing yielded *p*-values of 0.144 and 0.258, indicating no significant difference between the two methods tested. Kappa (κ) values of 0.881 and 1.00 reflected excellent inter-observer agreement. In the thalamus, positive findings were noted in 21.5% of FLAIR images and 32.3% of MinPE FLAIR/WAIR images, with a negligible difference (*p* = 0.166). The inter-observer agreement remained strong, with κ values of 0.916 and 0.939, confirming reliable lesion identification. In the internal capsule, the first observer identified positive findings in 15.4% of FLAIR images, which increased slightly to 18.5% with MinPE FLAIR/WAIR, with similar rates for the second observer. The chi-square tests yielded *p*-values of 0.640 and 0.818, again showing no significant difference. Kappa values of 0.946 and 1.00 indicated high inter-observer agreement. Overall, the total number of findings across all regions was 296 for FLAIR and 287 for MinPE FLAIR/WAIR for the first observer and 310 for FLAIR and 396 for MinPE FLAIR/WAIR for the second observer. These results suggest a consistent pattern of slightly higher detection with MinPE FLAIR/WAIR imaging. However, regional variations remained statistically insignificant, demonstrating a strong inter-observer reliability across the imaging methods (Fig. **5**).

In the midbrain, the first observer recorded positive findings in 13.8% of FLAIR images, increasing to 41.5% with the MinPE FLAIR/WAIR sequence. The second observer reported the same rates (13.8% for FLAIR and 41.5% for MinPE FLAIR/WAIR). The chi-square test yielded a *p*-value of 0.0004, indicating a statistically significant difference, suggesting that MinPE FLAIR/WAIR detected more midbrain findings than FLAIR alone. The inter-observer agreement was high, with kappa (κ) values of 0.814 for FLAIR and 1.00 for MinPE FLAIR/WAIR. In the pons, the first observer identified positive findings in 27.7% of FLAIR images, increasing to 72.3% with MinPE FLAIR/WAIR. The second observer noted a similar pattern, with positive findings increasing from 27.7% on FLAIR to 72.3% on MinPE FLAIR/WAIR. The chi-square test produced a *p*-value of 0.0001, indicating a significant difference between the two methods tested. Kappa values of 0.932 and 0.944 reflected strong inter-observer agreement.

In the medulla, positive findings were observed in 4.6% of FLAIR images, increasing markedly to 58.5% with MinPE FLAIR/WAIR. The chi-square test showed a highly significant difference (*p* = 0.0001), supporting the greater effectiveness of MinPE FLAIR/WAIR in detecting abnormalities in this region. Inter-observer agreement was perfect for FLAIR (κ = 1.00) and nearly perfect for MinPE FLAIR/WAIR (κ = 0.978). In the cerebellar peduncle, positive findings were noted in 21.5% of the FLAIR images, rising to 32.3% with MinPE FLAIR/WAIR. However, this difference was not statistically significant (*p* = 0.166). Kappa values of 0.874 and 0.939 indicated substantial inter-observer agreement (Table **3**).

**Fig. (6):**
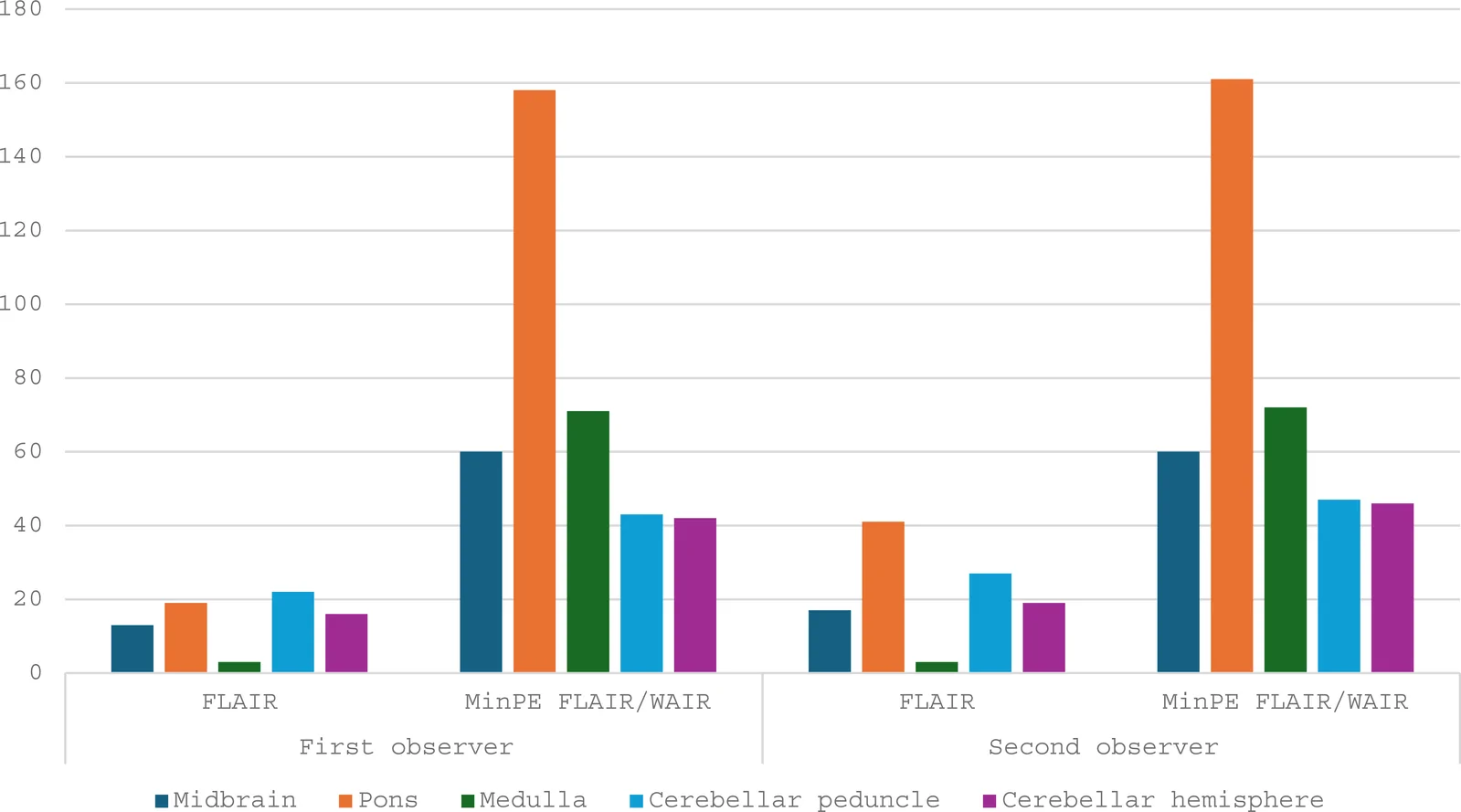
Comparison between the total number of findings in the Posterior Fossa in FLAIR and MinPE FLAIR/WAIR by the two observers and agreement between the two observers; the y-axis represents the number of detected MS lesions.

In the cerebellar hemisphere, the first observer noted positive findings in 16.9% of FLAIR images, increasing to 30.8% with MinPE FLAIR/WAIR sequences. The second observer reported similar results to the first. This difference was statistically significant (*p* = 0.043), suggesting that MinPE FLAIR/WAIR enhances lesion detection in this region of interest. The inter-observer agreement was high, with kappa (κ) values of 0.896 and 0.937. Overall, across all regions of the posterior fossa, the first observer recorded 91 findings on FLAIR images and 374 on MinPE FLAIR/WAIR images. The second observer reported 107 findings on FLAIR and 386 on MinPE FLAIR/WAIR sequences. These results consistently demonstrate higher lesion detection with MinPE FLAIR/WAIR, supporting its greater detection rates for identifying posterior fossa abnormalities, as further evidenced by the strong inter-observer agreement (Fig. **6**).

## 4 DISCUSSION

Multiple sclerosis (MS) is a chronic autoimmune disease characterized by inflammation, demyelination, and axonal damage in the central nervous system (CNS) [1, 2]. The diagnosis and management of MS rely heavily on neuroimaging techniques, particularly magnetic resonance imaging (MRI), which provides essential information about lesion burden, disease progression, and treatment response [7]. However, conventional MRI sequences, such as Fluid-Attenuated Inversion Recovery (FLAIR), have limitations in detecting certain types of MS lesions, especially those located in the subcortical white matter, thalamus, and brainstem [16]. These limitations underscore the need for improved imaging techniques to enhance the sensitivity and specificity of MS-lesion detection. Advanced techniques, such as Double Inversion Recovery (DIR), Magnetization Prepared 2 Rapid Acquisition Gradient Echoes (MP2RAGE), susceptibility-weighted imaging (SWI), Phase-Sensitive Inversion-Recovery (PSIR), and 3D inversion recovery ultrashort echo time (IR-UTE) sequences, have significantly improved the visualization and detection of cortical lesions. Nevertheless, their use in routine diagnostic and clinical trial protocols remains limited because of the prolonged acquisition time [30-35].

In this study, we evaluated the effectiveness of fused fluid-attenuated Inversion Recovery (FLAIR) and white matter-attenuated inversion recovery (WAIR) sequences (“MinPE FLAIR/WAIR”) in improving the detection of MS lesions. This method is based on suppressing dominant background tissue signals by combining FLAIR, which nulls the cerebrospinal fluid, and WAIR, which reduces the normal white matter signal. This voxel-wise minimum selection increases lesion conspicuousness by retaining the signal from MS lesions while minimizing the surrounding tissue signal. Our findings demonstrate promising results, indicating that “MinPE FLAIR/WAIR” images demonstrate enhanced lesion conspicuousness and higher lesion detection rates than conventional FLAIR sequences across various brain regions. The addition of a standard WAIR sequence, which is available in all MRI scanners, to the routine protocol adds only ~1.5 min to the total scan time.

One notable finding of our study was the increased detection of lesions in brain regions that are often missed or underestimated by conventional FLAIR imaging. The number and volume of lesions play significant roles in MS pathology and are associated with disease progression and cognitive impairment [2, 6, 7, 16]. Our findings suggest that MinPE FLAIR/WAIR may improve lesion detection in several brain regions compared with conventional 2D FLAIR. The mean number of lesions in MinPE FLAIR/WAIR was generally more than that seen in standard FLAIR within the supratentorial areas (eg, cortical gray matter, juxtacortical white matter, and basal ganglia). However, most of these differences were not statistically significant. The trend toward higher lesion counts indicates a potential added value that warrants further investigation in larger cohorts. Importantly, MinPE FLAIR/WAIR demonstrated a statistically significant improvement in detecting posterior fossa lesions, a region where subtle pathology can be challenging to visualize owing to anatomical constraints and CSF-related artifacts on standard imaging. This enhanced lesion conspicuousness in the infratentorial region highlights the clinical promise of MinPE sequences for a more comprehensive evaluation of the MS lesion burden [31, 36].

In the present study, the MinPE fusion technique substantially improved the conspicuousness of lesions across multiple brain regions, particularly in the posterior fossa. These findings are consistent with those of prior studies demonstrating that optimized image combination methods can enhance MS lesion detection beyond conventional FLAIR or T2-weighted sequences alone. Compared to other studies, our MinPE approach uniquely leverages information from both FLAIR (CSF suppression) and WAIR (white-matter signal attenuation), resulting in a synergistic enhancement of lesion-to-background contrast, especially in anatomically challenging regions, such as the midbrain, pons, and medulla. The inclusion of 95% confidence intervals (CIs) for the sensitivity estimates provided additional insight into the precision of our findings. The CIs indicate the range within which the true sensitivity of each imaging technique is likely to fall, allowing for a more nuanced interpretation of the comparative performance of MinPE FLAIR/WAIR and conventional FLAIR imaging. This is particularly important for assessing the robustness of the results, given the sample size and variability in the readings. The findings of our study have significant clinical implications for the diagnosis, management, and monitoring of MS [37], [18]. The improved lesion conspicuousness of MinPE FLAIR/WAIR images for detecting MS lesions may facilitate earlier identification of disease activity, more accurate assessment of disease burden, and improved monitoring of treatment response. Furthermore, the ability of MinPE FLAIR/WAIR to enhance lesion detection offers new opportunities to understand MS pathophysiology and develop targeted therapeutic interventions [26, 28, 31].

The results from our study were likewise consistent with previous studies that have evaluated how well different advanced imaging technologies, including double inversion recovery (DIR) or phase-sensitive inversion recovery (PSIR), can improve the identification of MS lesions [16, 38-40]. Many studies have also reported almost equal improvements in the ability to identify lesions, and that advanced MRI sequence imaging has added to or supported the ability to identify lesions and diagnose MS accurately. Again, there is no single best protocol, and the optimal imaging protocol for MS will depend on the stage of the disease, the type of lesion(s), and specific characteristics of the individual patient [16, 28, 38].

The addition of the WAIR sequence can be integrated into the standard MRI protocol with minimal disruption of the workflow. The primary requirement is the post-processing step required to generate the MinPE image contrast. This step is independent of the scanner and can be performed on an external workstation using routine image analysis software or can be fully automated within the institutional pipeline. Therefore, although a small amount of post-acquisition processing is necessary, the method can be readily incorporated into clinical practice without modifying the existing hardware or pulse-sequence structure. The proposed fusion approach is particularly advantageous in clinical settings where access to advanced imaging sequences is limited. By combining complementary information from routinely acquired MRI sequences, the method enhances lesion conspicuity without relying on specialized acquisitions such as double inversion recovery (DIR). This makes the approach especially suitable for older MRI scanners, where advanced imaging methods may not be available, and helps explain its improved performance compared with baseline techniques that depend on single-contrast information or specialized sequences.

## 5 LIMITATIONS AND CONSIDERATIONS

Despite these promising results, several limitations of our study should be considered when interpreting these findings. First, this study was conducted at a single center with a relatively small sample size, which may limit the generalizability of the results. Future multicenter studies with larger cohorts are needed to validate our findings and further assess the clinical utility of MinPE FLAIR/WAIR images in diverse patient populations. Additionally, the independent acquisition of FLAIR and WAIR sequences may allow for patient motion between acquisitions, potentially leading to image displacement and fusion errors, which can be corrected using dedicated registration techniques. In the current study, no quantitative metrics (*e.g.*, mutual information or Dice coefficient) were applied to validate coregistration accuracy; instead, coregistration quality was assessed using systematic visual inspection. Future studies should consider this limitation and compare our method with other established techniques, such as 2D and 3D DIR and PSIR, in the same patient cohort to enable a direct quantitative comparison of lesion conspicuity, sensitivity, and diagnostic value using quantitative metrics, such as SNR, CNR, or lesion-to-background ratio. Inter-reader variability may also arise due to lesion size, volume, or fragmentation, which may be influenced by co-registration accuracy or reader confidence. The present study was designed as an exploratory analysis, and several methodological aspects provide opportunities for future investigations. The application of multiple-comparison–aware statistical approaches and the inclusion of lesion-level specificity and false-positive metrics would strengthen the quantitative interpretation. The present study was designed as an exploratory analysis, and several methodological aspects provide opportunities for future investigations. The application of multiple-comparison–aware statistical approaches and the inclusion of lesion-level specificity and false-positive metrics would strengthen the quantitative interpretation. Future studies using consensus annotation or longitudinal imaging as reference standards may further clarify the clinical impact of improved lesion detection. The post-hoc power analysis should be interpreted with caution, as it was derived from observed effect sizes in regions demonstrating statistically significant differences. Consequently, the reported power does not imply uniform sensitivity to detect smaller differences across all brain regions. This limitation underscores the importance of cautious interpretation of non-significant findings and highlights the need for larger, prospective studies to evaluate more subtle regional differences. Finally, the retrospective study design and reliance on existing MRI datasets may have introduced selection bias and limited control over confounding variables. Prospective studies with standardized imaging protocols and longitudinal follow-ups are required to confirm the efficacy of MinPE FLAIR/WAIR in clinical practice.

## CONCLUSION

In conclusion, our study demonstrates that fused FLAIR and WAIR images, known as MinPE FLAIR/WAIR, demonstrate improved lesion conspicuousness and higher MS lesion detection rates compared with conventional FLAIR sequences. Improved lesion detection across various brain regions has important implications for the diagnosis, monitoring, and treatment planning of MS. Future research should aim to validate these findings in larger cohorts and explore the potential role of MinPE FLAIR/WAIR in predicting clinical outcomes and guiding therapeutic decisions in patients with MS.

## Figures and Tables

**Table 1:** Distribution of the studied patient group according to their demographic data.

**Variable**	**Number** **“n=65 patients”**	**Percent (%)**
**Sex** Male Female	18 47	27.7 72.3
**Age group** <25 years 25-30 30-40 >40	18 23 16 8	27.7 35.4 24.6 12.3
Range Mean ± S.D.	17-57 29.96 ± 2.11	

**Table 2:** Comparison of lesion detection between FLAIR and MinPE FLAIR/WAIR for both observers across different brain regions. The values represent the number and percentage of positive findings. Chi-square (X^2^) and *p*-values were used to assess the differences between sequences. *P*-values ≤ 0.05 were considered significant, and κ denotes inter-observer agreement for each imaging method.

**Brain Regions**	**Observer**	**FLAIR (n, %)**	**MinPE FLAIR/WAIR (n, %)**	**X^2^**	***P*-value**
**Cortical GM**	First	16 (24.6%)	24 (33.8%)	1.34	0.247
	Second	16 (24.6%)	24 (33.8%)	1.34	0.247
*κ*		1.00	1.00		
**Juxtacortical WM**	First	42 (43.1%)	52 (46.2%)	0.12	0.724
	Second	43 (43.1%)	53 (46.2%)	0.12	0.724
*κ*		0.971	0.973		
**Subcortical WM**	First	44 (52.3%)	58 (58.5%)	0.49	0.4803
	Second	45 (52.3%)	58 (58.5%)	0.49	0.4803
*κ*		0.972	1.00		
**Periventricular WM**	First	141 (87.7%)	170 (90.8%)	0.32	0.5715
	Second	148 (87.7%)	173 (90.8%)	0.32	0.5715
*κ*		0.936	0.968		
**Corpus callosum**	First	8 (12.3%)	10 (15.4%)	0.26	0.6115
	Second	9 (13.8%)	10 (15.4%)	0.06	0.804
*κ*		0.932	1.00		
**Basal ganglia**	First	4 (6.2%)	13 (13.8%)	2.15	0.144
	Second	5 (7.7%)	13 (13.8%)	1.28	0.258
*κ*		0.881	1.00		
**Thalamus**	First	24 (21.5%)	40 (32.3%)	1.92	0.166
	Second	26 (21.5%)	43 (32.3%)	1.92	0.166
*κ*		0.916	0.939		
**Internal capsule**	First	17 (15.4%)	20 (18.5%)	0.22	0.640
	Second	18 (16.9%)	20 (18.5%)	0.05	0.818
*κ*		0.946	1.00		
**Total findings**	First	296	387	—	—
	Second	310	394	—	—

**Table 3:** Comparison of lesion detection between FLAIR and MinPE FLAIR/WAIR for both observers across different Posterior Fossa regions. The values represent the number and percentage of positive findings. Chi-square (X^2^) and *p*-values were used to assess the differences between sequences. *P*-values ≤ 0.05 were considered significant, and κ denotes inter-observer agreement for each imaging method.

**Posterior Fossa Regions**	**Observer**	**FLAIR (n, %)**	**MinPE FLAIR/WAIR (n, %)**	**X^2^**	***P*-value**
**Midbrain**	First	13 (13.8%)	60 (41.5%)	12.45	**0.0004**
	Second	17 (13.8%)	60 (41.5%)	12.45	**0.0004**
*κ*		0.814	1.00		
**Pons**	First	19 (27.7%)	158 (72.3%)	25.88	**0.0001**
	Second	41 (27.7%)	161 (72.3%)	25.88	**0.0001**
*κ*		0.932	0.944		
**Medulla**	First	3 (4.6%)	71 (58.5%)	43.64	**0.0001**
	Second	3 (4.6%)	72 (58.5%)	43.64	**0.0001**
*κ*		1.00	0.978		
**Cerebellar peduncle**	First	22 (21.5%)	43 (32.3%)	1.92	0.166
	Second	27 (21.5%)	47 (32.3%)	1.92	0.166
*κ*		0.874	0.939		
**Cerebellar hemisphere**	First	16 (16.9%)	42 (30.8%)	3.43	**0.043**
	Second	19 (16.9%)	46 (30.8%)	3.43	**0.043**
*κ*		0.896	0.937		
**Total findings**	First	91	374	—	—
	Second	107	386	—	—

## Data Availability

The data of the current study are available from the corresponding author, [A.A], upon reasonable request.

## LIST OF ABBREVIATIONS

MS: Multiple sclerosis
FLAIR: Fluid-Attenuated Inversion Recovery
WAIR: White Matter Attenuated Inversion Recovery
MinPE: Minimum Pixel Value Extraction
WM: White Matter
GM: Gray Matter
DIR: Double Inversion Recovery
CSF: Cerebrospinal Fluid
CNS: Central Nervous System
SNR: Signal to Noise Ratio
RRMS: Relapsing Remitting Multiple Sclerosis
PPMS: Primary Progressive Multiple Sclerosis
EDSS: Expanded Disability Status Scale
MP2RAGE: Magnetization Prepared 2 Rapid Acquisition Gradient Echoes
PSIR: Phase-Sensitive Inversion-Recovery

## STANDARDS OF REPORTING

STARD guidelines were followed.

## References

[1] McGinley M.P., Goldschmidt C.H., Rae-Grant A.D. (2021). Diagnosis and treatment of multiple sclerosis.. JAMA.

[2] Dobson R., Giovannoni G. (2019). Multiple sclerosis – A review.. Eur. J. Neurol..

[3] Boutitah-Benyaich I., Eixarch H., Villacieros-Álvarez J., Hervera A., Cobo-Calvo Á., Montalban X., Espejo C. (2025). Multiple sclerosis: Molecular pathogenesis and therapeutic intervention.. Signal Transduct. Target. Ther..

[4] Walton C, King R, Rechtman L, Kaye W, Leray E, Marrie RA, Robertson N, La Rocca N, Uitdehaag B, van der Mei I, Wallin M, Helme A, Angood Napier C, Rijke N, Baneke P (2020). Rising prevalence of multiple sclerosis worldwide: Insights from the Atlas of MS, third edition.. Mult Scler.

[5] Vaughn C.B., Jakimovski D., Kavak K.S., Ramanathan M., Benedict R.H.B., Zivadinov R., Weinstock-Guttman B. (2019). Epidemiology and treatment of multiple sclerosis in elderly populations.. Nat. Rev. Neurol..

[6] Vidal-Jordana A., Montalban X. (2017). Multiple sclerosis.. Neuroimaging Clin. N. Am..

[7] Lassmann H. (2018). Multiple sclerosis pathology.. Cold Spring Harb. Perspect. Med..

[8] Baadsvik E.L., Weiger M., Froidevaux R., Faigle W., Ineichen B.V., Pruessmann K.P. (2023). Quantitative magnetic resonance mapping of the myelin bilayer reflects pathology in multiple sclerosis brain tissue.. Sci. Adv..

[9] Faissner S., Plemel J.R., Gold R., Yong V.W. (2019). Progressive multiple sclerosis: From pathophysiology to therapeutic strategies.. Nat. Rev. Drug Discov..

[10] Filippi M., Brück W., Chard D., Fazekas F., Geurts J.J.G., Enzinger C., Hametner S., Kuhlmann T., Preziosa P., Rovira À., Schmierer K., Stadelmann C., Rocca M.A., Attendees of the Correlation between Pathological and MRI findings in MS workshop (2019). Association between pathological and MRI findings in multiple sclerosis.. Lancet Neurol..

[11] Williams K.L., Brauer S.G. (2022). Walking impairment in patients with multiple sclerosis: The impact of complex motor and non-motor symptoms across the disability spectrum.. Aust. J. Gen. Pract..

[12] Rocca M.A., Preziosa P., Barkhof F., Brownlee W., Calabrese M., De Stefano N., Granziera C., Ropele S., Toosy A.T., Vidal-Jordana À., Di Filippo M., Filippi M. (2024). Current and future role of MRI in the diagnosis and prognosis of multiple sclerosis.. Lancet Reg. Health Eur..

[13] Geraldes R., Ciccarelli O., Barkhof F., De Stefano N., Enzinger C., Filippi M., Hofer M., Paul F., Preziosa P., Rovira A., DeLuca G.C., Kappos L., Yousry T., Fazekas F., Frederiksen J., Gasperini C., Sastre-Garriga J., Evangelou N., Jacqueline Palace on behalf of the MAGNIMS study group (2018). Erratum: The current role of MRI in differentiating multiple sclerosis from its imaging mimics.. Nat. Rev. Neurol..

[14] Weidauer S., Raab P., Hattingen E. (2021). Diagnostic approach in multiple sclerosis with MRI: An update.. Clin. Imaging.

[15] Righart R., Biberacher V., Jonkman L.E., Klaver R., Schmidt P., Buck D., Berthele A., Kirschke J.S., Zimmer C., Hemmer B., Geurts J.J.G., Mühlau M. (2017). Cortical pathology in multiple sclerosis detected by the T 1/ T 2‐weighted ratio from routine magnetic resonance imaging.. Ann. Neurol..

[16] Almutairi AD, Mahmud R, Suppiah S, Hassan HA (2019). Accuracy of MRI sequences in detecting multiple sclerosis (ms) lesions: A systematic review.. Adv Biosci Clin Med.

[17] Almutairi A.D., Hassan H.A., Suppiah S. (2020). Lesion load assessment among multiple sclerosis patient using DIR, FLAIR, and T2WI sequences.. Egypt. J. Radiol. Nucl. Med..

[18] Essel R.R., Krieger B., Bellenberg B., Müller D., Ladopoulos T., Gold R., Schneider R., Lukas C. (2025). Lesion assessment in multiple sclerosis: A comparison between synthetic and conventional fluid-attenuated inversion recovery imaging.. Front. Neurol..

[19] Barkhof F., Reich D.S., Oh J., Rocca M.A., Li D.K.B., Sati P., Azevedo C.J., Bagnato F., Calabresi P.A., Ciccarelli O., Dwyer M.G., DeLuca G.C., De Stefano N., Enzinger C., Filippi M., Granziera C., Halper J., Henry R.G., Gasperini C., Gauthier S., Kappos L., Laule C., Newsome S.D., Montalban X., Morrow S.A., Schoonheim M.M., Sicotte N., Toosy A., Wilken J., Yousry T., Sastre-Garriga J., Traboulsee A., Ontaneda D., Rovira À., Magnetic Resonance Imaging Network in Multiple Sclerosis, Consortium of Multiple Sclerosis Centers, North American Imaging in Multiple Sclerosis Cooperative MRI guidelines working group (2025). 2024 MAGNIMS–CMSC–NAIMS consensus recommendations on the use of MRI for the diagnosis of multiple sclerosis.. Lancet Neurol..

[20] Motegi S., Shimada T., Hayashi N., Nagase H., Taketomi-Takahashi A., Tsushima Y. (2017). Double inversion recovery imaging of the brain: Deriving the most relevant sequence through real images.. Radiological Phys. Technol..

[21] Mostardeiro T.R., Schmitt L.G., de Campos F.T.X., Xi Y., Brondani Torri G., Carvalho B.M., Feltrin F.S. (2025). Multiple sclerosis lesion detection with 3D double inversion recovery (DIR) as compared to 3D fluid low attenuation inversion recovery (T2-FLAIR): A systematic review and meta-analysis.. Mult. Scler. Relat. Disord..

[22] Alharbi K.O., Abujamea A.H., Alomair O.I., Alsakkaf H.M., Alharbi A.A., Alghamdi S.A., Alharbi A.G. (2022). Improving cervical spinal cord lesion detection in multiple sclerosis using filtered fused proton density-T2 weighted images.. Acta Radiol. Open.

[23] Lespagnol M., Massire A., Megdiche I., Lespagnol F., Brugières P., Créange A., Stemmer A., Bapst B. (2023). Improved detection of juxtacortical lesions using highly accelerated double inversion-recovery MRI in patients with multiple sclerosis.. Diagn. Interv. Imaging.

[24] Tomura N., Saginoya T., Sanpei T., Konno T., Fujihara K. (2023). Contrast-enhanced double inversion recovery sequence for patients with multiple sclerosis: Feasibility of subtraction images between pre- and post-contrast images.. Acta Radiol..

[25] Murumkar V., Priyadarshini Baishya P., Kulanthaivelu K., Saini J., Manjunath N., Kumar Gupta R. (2022). Comparison of 3D double inversion recovery (DIR) *versus* 3D fluid attenuated inversion recovery (FLAIR) in precise diagnosis of acute optic neuritis.. Eur. J. Radiol..

[26] Elnekeidy A.M., Kamal M.A., Elfatatry A.M., Elskeikh M.L. (2014). Added value of double inversion recovery magnetic resonance sequence in detection of cortical and white matter brain lesions in multiple sclerosis.. Egypt. J. Radiol. Nucl. Med..

[27] Sakoda K., Baba S. (2024). Technical Note: Novel imaging method to obtain gray matter-attenuated inversion recovery image using low-field magnetic resonance imaging systems.. Radiography.

[28] Costagli M., Lapucci C., Zacà D., Bruschi N., Schiavi S., Castellan L., Stemmer A., Roccatagliata L., Inglese M. (2022). Improved detection of multiple sclerosis lesions with T2‐prepared double inversion recovery at 3T.. J. Neuroimaging.

[29] Chard D.T. (2025). Diagnosing multiple sclerosis.. Neurology.

[30] Sedaghat S., Jang H., Ma Y., Afsahi A.M., Reichardt B., Corey-Bloom J., Du J. (2023). Clinical evaluation of white matter lesions on 3D inversion recovery ultrashort echo time MRI in multiple sclerosis.. Quant. Imaging Med. Surg..

[31] Schmidt C., Hattingen E., Faehndrich J., Jurcoane A., Porto L. (2012). Detectability of multiple sclerosis lesions with 3 T MRI: A comparison of proton density-weighted and FLAIR sequences.. J. Neuroradiol..

[32] Spini M., Choi S., Harrison D.M. (2020). 7T MPFLAIR *versus* MP2RAGE for quantifying lesion volume in multiple sclerosis.. J. Neuroimaging.

[33] Bouman P.M., Noteboom S., Nobrega Santos F.A., Beck E.S., Bliault G., Castellaro M., Calabrese M., Chard D.T., Eichinger P., Filippi M., Inglese M., Lapucci C., Marciniak A., Moraal B., Morales Pinzon A., Mühlau M., Preziosa P., Reich D.S., Rocca M.A., Schoonheim M.M., Twisk J.W.R., Wiestler B., Jonkman L.E., Guttmann C.R.G., Geurts J.J.G., Steenwijk M.D. (2023). Multicenter evaluation of ai-generated DIR and PSIR for cortical and juxtacortical multiple sclerosis lesion detection.. Radiology.

[34] Jang H., Ma Y.J., Chang E.Y., Fazeli S., Lee R.R., Lombardi A.F., Bydder G.M., Corey-Bloom J., Du J. (2021). Inversion recovery ultrashort te mr imaging of myelin is significantly correlated with disability in patients with multiple sclerosis.. AJNR Am. J. Neuroradiol..

[35] Afkandeh R., Abedi I., Zamanian M. (2024). Detection of multiple sclerosis lesions by susceptibility-weighted imaging—A systematic review and meta-analyses.. Clin. Radiol..

[36] Jehna M., Pirpamer L., Khalil M., Fuchs S., Ropele S., Langkammer C., Pichler A., Stulnig F., Deutschmann H., Fazekas F., Enzinger C. (2015). Periventricular lesions correlate with cortical thinning in multiple sclerosis.. Ann. Neurol..

[37] Bachmann R., Reilmann R., Schwindt W., Kugel H., Heindel W., Krämer S. (2006). FLAIR imaging for multiple sclerosis: A comparative MR study at 1.5 and 3.0 Tesla.. Eur. Radiol..

[38] Abidi Z., Faeghi F., Mardanshahi Z., Mortazavi H. (2017). Assessment of the diagnostic accuracy of double inversion recovery sequence compared with FLAIR and T2W_TSE in detection of cerebral multiple sclerosis lesions.. Electron. Physician.

[39] Favaretto A., Poggiali D., Lazzarotto A., Rolma G., Causin F., Gallo P. (2015). The parallel analysis of phase sensitive inversion recovery (psir) and double inversion recovery (DIR) images significantly improves the detection of cortical lesions in multiple sclerosis (MS) since clinical onset.. PLoS One.

[40] Sedaghat S., Jang H., Athertya J.S., Groezinger M., Corey-Bloom J., Du J. (2023). The signal intensity variation of multiple sclerosis (MS) lesions on magnetic resonance imaging (MRI) as a potential biomarker for patients’ disability: A feasibility study.. Front. Neurosci..

