# Supplementary material for: Enhancing the Visibility of Multiple Sclerosis Lesions using Fused Images of Fluid Attenuated Inversion Recovery (FLAIR) and white matter Attenuated Inversion Recovery (WAIR) MRI Sequences
Source: Curr Med Imaging. 2026 Apr 8;22:E15734056437290. doi: 10.2174/0115734056437290260309135652 (PMC13584081; doi:10.2174/0115734056437290260309135652)
Supplement: Supplementary file 1 [file CMIM-22-E15734056437290_SD1.pdf]

1875-6603/26 *Send Orders for Reprints to reprints@benthamscience.net*

BENTHAM SCIENCE

CrossMark

Current Medical Imaging

Content list available at: https://benthamscience.com/journals/cmir

Supplementary Material

Enhancing the Visibility of Multiple Sclerosis Lesions using Fused Images of Fluid Attenuated Inversion Recovery (FLAIR) and white matter Attenuated Inversion Recovery (WAIR) MRI Sequences

Khalid Alanazi[1,3,#], Abdullah Abujamea[1,2,*,#], Muhammad Fahim Amjad[3], Almutairi Fahad[1] and Ali Abdu[1]

[1]*Department of Radiology and Medical Imaging, King Saud University Medical City, Riyadh, Saudi Arabia*
[2]*Department of Radiology and Medical Imaging, College of Medicine, King Saud University, Riyadh, Saudi Arabia*
[3]*Department of Radiology, King Khalid Hospital, Tabuk, Saudi Arabia*

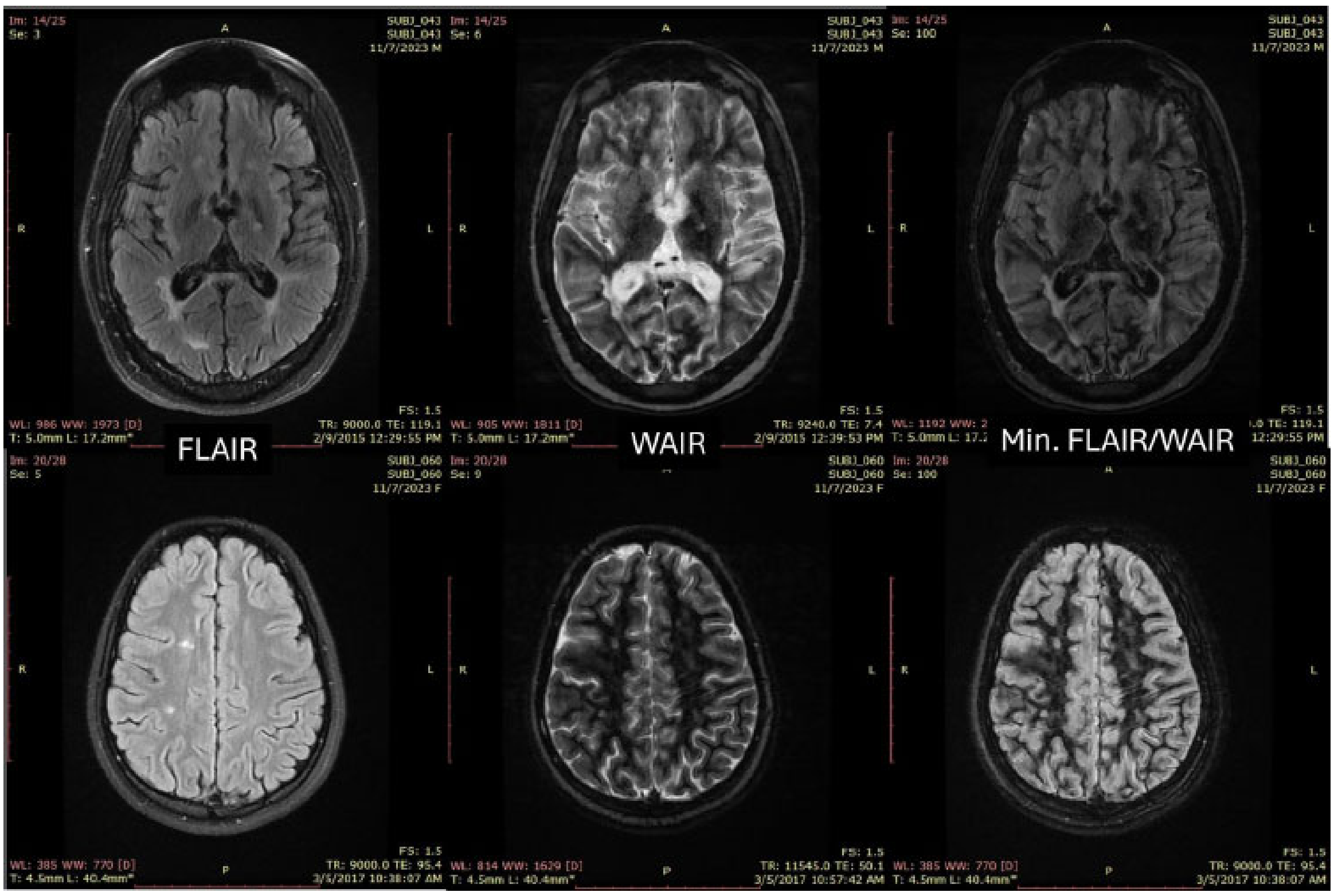

Fig. (S1). Example rejected case as a result of misregistration because of patient movement between the two acquisitions of FLAIR and WAIR which result in a sever image distortion.

© 2026 The Author(s). Published by Bentham Science Publisher.

This is an open access article distributed under the terms of the Creative Commons Attribution 4.0 International Public License (CC-BY 4.0), a copy of which is available at: https://creativecommons.org/licenses/by/4.0/legalcode. This license permits unrestricted use, distribution, and reproduction in any medium, provided the original author and source are credited.

HOW TO CITE:
Alanazi K, Abujamea A, Amjad M, Fahad A, Abdu A. Enhancing the Visibility of Multiple Sclerosis Lesions using Fused Images of Fluid Attenuated Inversion Recovery (FLAIR) and white matter Attenuated Inversion Recovery (WAIR) MRI Sequences. Curr Med Imaging, 2026; 22: e15734056437290.
http://dx.doi.org/10.2174/0115734056437290260309135652
